# 

**Published:** 1902-11-01

**Authors:** 


### NOTICE TO CORRESPONDENTS.

All MSS., Letters, Books for Review, and other matters intended for the Editor should be addressed THE EDITOR, "THE HOSPITAL," THE HOSPITAL BUILDING, 28 & 29 SOUTHAMPTON STREET, STRAND, W.C.

The Editor cannot undertake to return rejected MS., even when accompanied by stamped directed envelope.

**Notice to Advertisers and Subscribers.**

All Advertisements, Orders for Copies of THE HOSPITAL, and Business Communications should be addressed to **THE MANAGER, (Not to the Editor)**, THE HOSPITAL, 28 & 29 Southampton Street, Strand, London, W.C.

The Cost of Subscription, post free, is as follows.—Per week, 2½d.; per quarter, 2s. 8d.; per half-year, 5s. 3d.; per annum, 10s. 6d. Foreign:—Per annum, 15s. 2d., payable, in all cases, in advance.

**NOTICE.—Vol. XXXII. of "The Hospital" (April to September 1902) in handsome cloth binding will shortly be on sale at the office of this Journal, price, 7s. 6d. Cases for binding the Numbers contained in this Volume 1s. 6d. Index to Vol. XXXII., 2½d., post free.**

**The Monthly Part for November is now ready. Price 6d., by post 8d.**

### BOOKS RECEIVED.

BAILLIÈRE, TINDALL AND COX.

"A Manual of Surgery for Students and Practitioners." By William Rose, M.B.Lond., F.R.C.S., and Albert Carless, M.S.Lond., F.R.C.S. Fifth Edition.

W. B. CLIVE.

"Advanced Hygiene." By Alfred E. Ikin, B.Sc. (Hons.) Lond., L.C.P.; and Robert A. Lyster, M.B., B.Ch., B.Sc.Lond., D.P.H.

JOHN BALE, SONS AND DANIELSSON.

"The Ambulance in Civil Life." By Reginald Harrison, F.R.C.S.

### THE STUDENT OF MEDICINE.

### APPOINTMENTS.

**Sydney Chalmers Allen**, M.B., B.Sc., appointed Assistant Medical Officer of the Lunatic Asylum at Seacliff, New Zealand. **W. Benthall**, M.B.Camb, M.R.C.S.Eng., appointed Honorary Consulting Surgeon to the Derbyshire Infirmary. **Robert Burnet**, M.Sc., M.B., Ch.B.Vict., appointed District Medical Officer to the Parish of Birmingham. **William J. Caie**, M.A., M.B., Ch.B.Aberd., appointed Public Vaccinator for the Borough of Bury St. Edmunds. **John T. Chapman**, L.R.C.P., M.R.C.S.Edin., appointed Health Officer for Eltham, Eastern Riding, Victoria. **Harold L. Cummings**, L.R.C.P., M.R.C.S.Eng., appointed Government Medical Officer and Vaccinator at Braidwood, New South Wales. **L. E. Ellis**, M.B., M.Ch.Syd., appointed Government Medical Officer and Vaccinator at Manila, New South Wales. **Edward Elphick**, L.R.C.P.Lond., M.R.C.S.Eng., appointed District Medical Officer at Newcastle and Public Vaccinator for the Urban and Suburban Districts of Newcastle and Rural District of Toodvav, Western Australia. **Leslie Thomas Gillespie**, M.B., appointed Health Officer for the Shire of Tungamob, Victoria. **Mabel Jessie Graham**, M.B.Syd., appointed Government Vaccinator at Petersham, New South Wales. **Allan Godwin Jackson**, M.D., appointed Health Officer for the North and West Ridings of the Shire of Ripon, Victoria. **J. C. Jellett**, L.R.C.P.&S.I., appointed District Medical Officer of the Billesdon Union. **R. O. Jones**, L.R.C.P.&S.Edin., appointed District Medical Officer of the Kingsbridge Union. **R. G. Kirton**, M.D.Lond., D.P.H., appointed Principal Medical Officer to the Prisons Department, Cairo, Egypt. **Andrew B. Morris**, L.R.C.P.&S.Irel., appointed Health Officer for the District of St. Helens, Tasmania. **James Ratcliff-Gaylard**, M.D.Brux., L.R.C.P., L.R.C.S., L.F.P.S.Glas., appointed Certifying Factory Surgeon for the Truro District of the county of Cornwall; Medical Officer of the Truro Workhouse, and St. Clements District; and Public Vaccinator of the St. Clements and East Kenwyn Districts of the Truro Union. **James M. Roe**, M.B.Syd., appointed Medical Officer at Tenningering (Mount Perry), Queensland. **F. B. Thornton**, M.B., appointed Honorary Physician to the Derbyshire Infirmary. **C. A. Wilkinson**, L.R.C.P.&S.Edin., L.F.P.S.Glas., appointed Surgeon for the Scarborough District of the North-Eastern Railway Company.

### "The Hospital" Institutional Library.

"Hospitals and Asylums of the World." 4 Vols., with a Portfolio of Plans, complete £12 12s.

"Burdett's Hospitals and Charities, 1902." 5s.

"Cottage Hospitals, General, Fever, and Convalescent." 10s. 6d.

"Hospital Expenditure: The Commissariat." 2s. 6d.

"A Legal Handbook for the Use of Hospital Authorities." 2s. 6d.

"The Cottage Hospital Case Book or Register of Patients." 10s. and 12s. 6d.

"The Furnishing and Appliances of a Cottage Hospital." 6d.

All these are published by the Scientific Press, Ltd., and may be obtained through any bookseller, or direct from the publishers, 28 and 29 Southampton Street, Strand, London, W.C.

